# Deimplementation of Polycythemia Screening in Asymptomatic Infants in a Level 1 Nursery

**DOI:** 10.1097/pq9.0000000000000533

**Published:** 2022-03-30

**Authors:** Scarlett C. Johnson, Elizabeth Bigus, Patricia L. Thompson, David G. Bundy, Michelle I. Amaya

**Affiliations:** From the Department of Pediatrics, Medical University of South Carolina, Charleston, SC.

## Abstract

Polycythemia (venous hematocrit >65%) is rare in healthy newborns (incidence: 0.4%–5%), with serious outcomes (stroke, bowel ischemia) of unknown incidence in asymptomatic infants. No national guidelines address screening or management of asymptomatic infants with polycythemia. Our nursery screened “high risk” (HR) newborns (small for gestational age, large for gestational age, twin, infant of diabetic mother) with poor adherence and low yield. We aimed to decrease polycythemia screening of asymptomatic HR infants by 80% within 6 months.

**Methods:**

We conducted an improvement project at a tertiary children’s hospital using the Model for Improvement. Eligible infants had an HR ICD-10 code on their problem list, were asymptomatic, over 35 weeks gestational age, and remained in the nursery for >6 hrs. Interventions included discontinuation of prior protocol, education for staff, bimonthly feedback on project performance, and visual reminders. Our primary outcome measure was the proportion of asymptomatic infants who received a hematocrit screen. Secondary measures were screening costs. Balancing measures were the length of stay, detected/symptomatic polycythemia, transfers to ICU/wards, and readmissions within 1 week of discharge.

**Results:**

The Nursery unit screened 80% of HR infants at baseline. This decreased to 7.3% after PDSA1, 0% after PDSA2, and 1% after PDSA3. There was no symptomatic polycythemia or statistically significant increase in readmissions/transfers. One month of monitoring revealed persistent changes.

**Conclusion:**

Simple quality improvement interventions such as education, reminders, and feedback can facilitate the deimplementation of low-value practices.

## INTRODUCTION

Five percent of healthcare expenditures involve tests and procedures that do not improve patient outcomes.^1^ Inpatient, well-newborn care is one of the fastest-growing areas of spending.^2^ The American College of Physicians defines high-value care as that in which outcomes and patient experience outweigh potential harm and invested resources.^1^ Deimplementation involves identifying low-value services, facilitating removal, evaluating outcomes, and sustaining results.^3^ Multifaceted approaches appear to be most successful and can include evidence-based practice standardization, restructuring/limiting associated funding, audit and feedback, clinical decision tools, as well as family/provider education.^3–6^ Such efforts result in substantial cost savings.^5,6^

One area of potential low-value care is screening for polycythemia in asymptomatic infants. Polycythemia is a venous hematocrit greater than 65%.^7,8^ The definition and the management of polycythemia are empirical and not evidence-based.^9^ Incidence of polycythemia in healthy term newborns is 0.4%–5%.^8–10^ Hematocrit peaks at 2 hours of life (HOL).^9,10^ The clinical concern is that increased hematocrit will increase the viscosity of the blood and decrease blood flow to vital organs. This change can result in hypoxia, acidosis, microthrombi, renal vein thrombosis, necrotizing enterocolitis, stroke, and hypoglycemia.^8,9^ The literature is sparse and outdated. Still, up to 47% of infants who meet the definition of polycythemia demonstrate symptoms. However, the prevalence of complications in asymptomatic infants is unknown.^8^ The majority of polycythemic infants are asymptomatic; when present, symptoms are nonspecific and include ruddy complexion, irritability, jitteriness, tremors, feeding difficulties, jaundice, apnea, cyanosis, respiratory distress, seizures, and lethargy. There is no human data that demonstrate blood viscosity exponentially increases above a hematocrit of 65%.^1,10^ We cannot accurately predict which infants will go on to be symptomatic or develop serious complications. Limited literature suggests asymptomatic or mildly symptomatic polycythemic infants do not benefit from exchange transfusion^11^ or even intravenous fluid hydration.^12^ Given the unknown natural history, the inability to confidently recognize a latent or early symptomatic stage, an uncertain optimal hematocrit threshold, and an unknown best practice for treatment of recognized disease, this practice does not meet criteria of a good screening test as defined by Wilson and Jungner and is an example of low-value care.^13^ False positives can result in heightened anxiety and further unnecessary interventions.^4^

Our institution’s nursery had a clinical protocol in place since 1997 (last revised in 2007) to screen for polycythemia in newborns considered to be at a higher risk for polycythemia: infant of a diabetic mother (IDM), maternal congenital heart disease, large for gestational age (LGA), small for gestational age (SGA), out of hospital deliveries, multiple gestations, and greater than 42 weeks gestation. We conducted a chart review of 1.5 years (n = 196) of Medical University of South Carolina (MUSC) Pediatric Hospital Information System (PHIS) data (after systematic institution of delayed cord clamping of 30–60 seconds).^14^ The nursery screened 80% of the at-risk group during their nursery stay (only 47% within 6 HOL). Fourteen infants had a heel stick hematocrit of >65%, with only one of those having a venous hematocrit of >65%. This IDM infant had a venous hematocrit of 73.8 at 8 HOL. He was transferred to the NICU, where the next hematocrit was already trending down on arrival but still >65%; so IV fluids were initiated. Before transfer, he had intermittent tachypnea and one episode of hypoglycemia on glucose screening that resolved with feeding. The patient was transferred back to the nursery after 12 hours of IV fluids and discharged home after another 24 hours of feeding and routine care. There were no other adverse outcomes. Therefore, we aimed to use quality improvement methodology to decrease hematocrit screening in asymptomatic HR infants in the Level 1 nursery by 80% within 6 months.

## METHODS

### Context

MUSC is a tertiary children’s hospital. Our level 1 nursery has one team at one location, caring for >2400 admissions per year and carrying an average daily census of 14.3 (2020 data).

### Planning

**Fig. 1.**
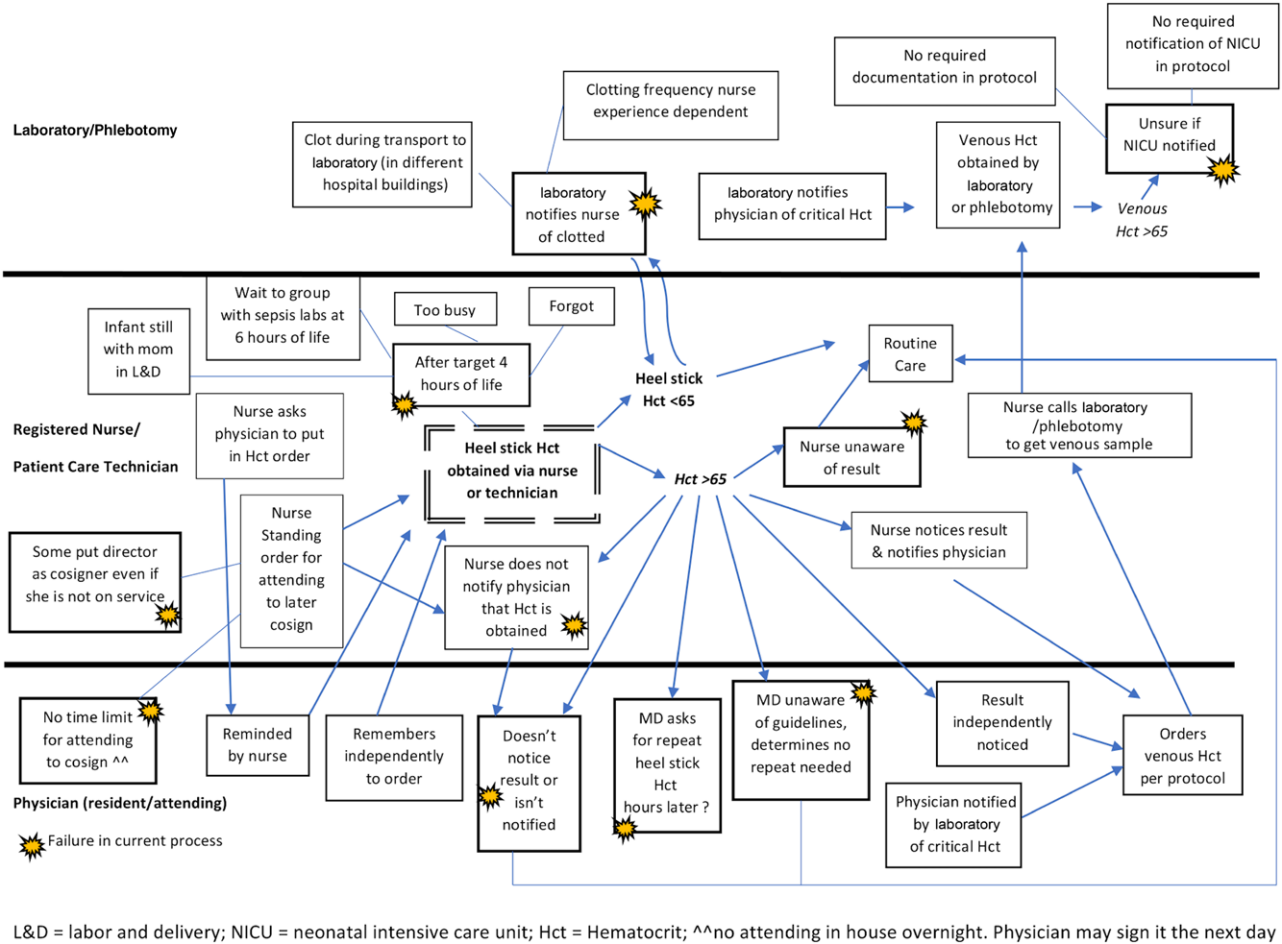
Process map of hematocrit screening within MUSC Level 1 nursery. Hct, Hematocrit; PCY, polycythemia; CBC, complete blood count; L&D, Labor and Delivery. **Laboratory does not notify nursing staff, and laboratory does not always notify MD.

**Fig. 2.**
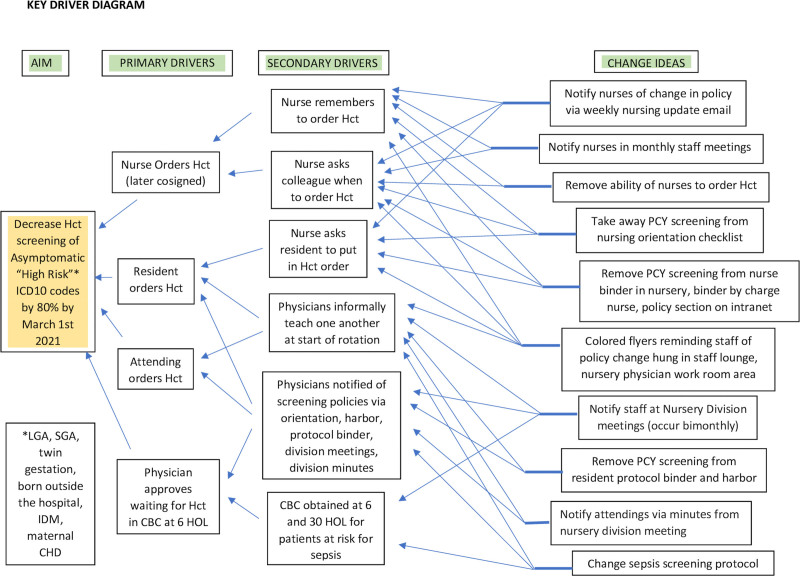
Key driver diagram of change ideas to remove hematocrit screening within MUSC Level 1 nursery. IDM, infant of diabetic mother, CHD, congenital heart disease, CBC, complete blood count.

The team included a pediatric hospital medicine fellow, nursery medical director, pediatric resident, and charge nurse. Conversations with a convenience sample of 15 nurses and technicians and five residents over several weeks aided in creating a process map. They revealed a lack of awareness about the specifics of the screening guidelines or where to find them. Nurses obtained hematocrits via standing order and later cosigned by the attending, who was not in-house overnight. A REDCap survey of nursery attending experience, understanding, and perspective revealed that half of the respondents wanted to continue using the guidelines and that in 6/8, knowing an asymptomatic infant had polycythemia would prompt additional action, including NICU consult for intravenous fluids, formula supplementation, closer monitoring of bilirubin levels, and glucose levels as well as prolonging admission for additional observation. We discussed the current literature revealing a lack of evidence and results of the 1.5-year PHIS chart review at a nursery attending division meeting. We selected initial interventions from our process map (Fig. 1) and key driver diagram (Fig. 2). This study was reviewed and designated as not human subjects research. Therefore, the study did not require review and approval by the MUSC Institutional Review Board.

### Establishing a Baseline

We selected a smaller window (7/1/19–9/30/19) of the original PHIS analysis [the most common ICD-10 codes corresponding to LGA (P08.1), SGA (P05.1), IDM (P70.1), and twin (Z37.9, Z38.32, Z37.2)] to establish a baseline before beginning the PDSA cycles. Eligible infants were then manually chart reviewed.

### Improvement Activities (Fig. 3)

#### PDSA 1: Remove Protocol and Education

We removed the protocol from the intranet as well as the resident resource binder. It was also taken out from the nurse orientation process.

**Fig. 3.**
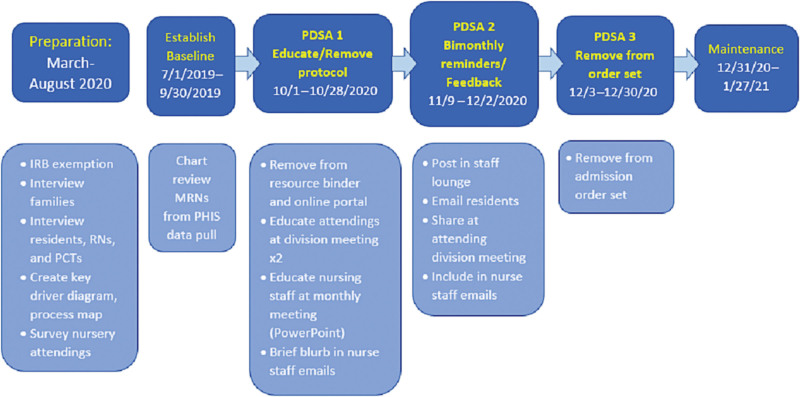
Project outline and timeline. PDSA, Plan do study act, IRB, Institutional review board. Education provided during September specified start date of 10/1/2020 for removal of the protocol.

The team presented an education session for both nursing staff and attendings at separate monthly meetings. The presentation briefly reviewed polycythemia and its symptoms, our reasoning for the deimplementation of screening, and clarification of the protocol from now on. This information was emailed out twice to both the nursing staff and the categorical pediatric residents. We created a one-page educational handout discussing polycythemia, obtaining a hematocrit based on clinical concerns, and delineating documentation, communication, and follow-up expectations for insertion in the resident educational binder and intranet website. We requested the staff to document any hematocrit obtained on symptomatic patients through a “progress note” in the electronic health record. Documentation directions included information regarding risk factor(s), the HOL, symptoms/signs noted, and steps taken, including hematocrit levels and any conversation with or recommendations from the NICU.

#### PDSA 2: Feedback and Reminders

We emailed bimonthly feedback regarding division-wide screening numbers to nursing, attending, and resident staff and posted visual flyers in the staff lounges. This information included the objective and rationale for deimplementation.

#### PDSA 3: Remove Hematocrit Order from the Order Set

IT (information technologists) removed the screening hematocrit order from the newborn nursery admission order set within the electronic medical record system (where it was listed but not preselected). Our team notified the nursing staff of this change to the order set in through a weekly newsletter.

### Eligibility Criteria

Eligible infants were SGA, LGA, IDM, or twin and admitted to the level 1 nursery, >35 weeks gestation, and remained in the nursery for > 6 hours before any potential transfer. We did not want to interfere with obtaining hematocrits on symptomatic polycythemic infants; so infants documented as symptomatic with a venous hematocrit of >65% were deemed ineligible. If there was no documentation of symptoms, then it was deemed a screen on an asymptomatic infant.

### PDSA Data-collection Process

For each PDSA cycle, one individual scanned the problem list of each nursery infant twice a day to identify high-risk infants. These medical record numbers *were tracked* and later chart-reviewed at the middle and end of the PDSA cycle. The fellow and resident chart reviewed the following information: gestational age, risk factor(s), obtainment of hematocrit laboratory, hematocrit value, HOL of the laboratory, obtainment of a confirmatory venous sample, and presence of polycythemia symptoms (in progress notes, discharge summaries, or transfer/accept notes as well as readmissions within 1 week of discharge).

### Measures

Our primary measure was the proportion of eligible high-risk infants screened for polycythemia with a hematocrit. Balance measures included length of stay (LOS), transfers, readmissions within 1 week of discharge, and symptomatic polycythemia cases. LOS was selected under the reasoning that not having objective laboratory data may result in prolonged patient observation.

### Analysis

A run chart evaluated the primary measure. Rules for determining a special cause variation were utilized. We ran Fisher exact and *t* test analyses on the primary outcome and balance measures via Excel and SAS version 9.4 (SAS Inc, Cary, N.C.). We compared each PDSA cycle versus the baseline 3-month period.

## RESULTS

### Baseline Period

**Fig. 4.**
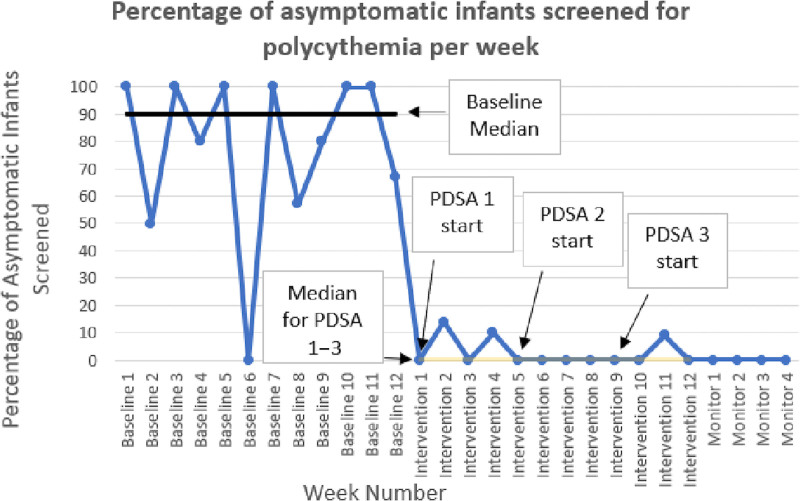
Run chart of the percentage of asymptomatic infants screened for polycythemia by week. Baseline (12 weeks): 7/8–9/29/19. PDSA1: 10/1–10/28/20. PDSA 2: 11/9–12/3/20. PDSA 3: 12/4–12/30/20. Monitoring period (no intervention): 12/31/20–1/27/21.

We chart reviewed a 3-month subset of the original PHIS data pull more thoroughly as our baseline (7/1/19−9/30/19, N = 40). Nurses screened 80% of eligible high-risk infants with a hematocrit (Fig. 4, Table 1). The screened infants were mostly term (67.5%), and a majority of them were LGA (52.5%) and IDM (30%). The average LOS for those who remained in the nursery to discharge (87.5%) was 2.5 days (SD 1.1). Two of the 32 screened infants had a positive heel stick screen with one of the two follow-up venous hematocrits > 65, prompting transfer to the NICU for intravenous fluids. There was one readmission (for hyperbilirubinemia) out of the 40 infants.

**Table 1.** Results of Baseline, PDSA 1, PDSA 2, PDSA 3, and Monitoring Period.

	Baseline 3 Months 7/1−9/30/19	PDSA 1 10/1−10/28/20	PDSA 2 11/9−12/2/20	PDSA 3 12/3−12/30/20	Monitoring 12/31/20−1/27/21
No. days in cycle	92	28	24	28	28
Intervention	N/A	Education and remove protocol	Bimonthly reminders/ results and feedback	Removed hematocrit from order set	N/A
Eligible high-risk* infants	40	41	30	33	43
Term† N(%)	27 (67.5)	30 (73)	23 (76.7)	25 (75.8)	40 (93)
SGA N(%)	5 (12.5)	11 (26.8)	9 (30)	8 (24.2)	13 (30.2)
LGA N(%)	21 (52.5)	9 (22)	3 (10)	9 (27.2)	16 (37.2)
IDM N(%)	12 (30)	20 (48.8)	13 (43.3)	16 (48.4)	12 (27.9)
Twin N(%)	9 (22.5)	9 (22)	8 (26.7)	4 (12.1)	4 (9.3)
2 or more risk factors N(%)	6 (15)	7 (17)	3 (10)	4 (12.1)	2 (4.6)
Mean LOS days (SD) nursery/ all eligible	2.5 (1.1)/ 3.1 (2.4)	2.1(0.7)^‡^/ 2.7(2.4)	2.1 (0.7)^‡^/ 3.1 (5.8)	2.1 (0.9)^‡^/ 2.8 (1.5)	2.1(0.8)^‡^/ 2.2(0.9)
Hematocrit screened N (%)	32 (80)	3 (7.3)^‡^	0^‡^	1 (3)^‡^	0^‡^
Positive screens (heel stick hematocrit > 65)	2	2	N/A	0	N/A
Polycythemia (venous hematocrit > 65)	1	1	N/A	0	N/A
Transfer to NICU for hypoglycemia N(%)	2 (5)	2 (4.9)	3 (10)	4 (12.1)	3 (7.0)
Transfers for other reasons	2	4	0	4	0
Transfers to NICU for PCY	1	0	0	0	0
Readmitted within 1 week of discharge	1 (hyperbilirubinemia, no polycythemia)	^1 (hyperbilirubinemia)	^2 (hyperbilirubinemia and sepsis evaluation)	^2 (hypoxia and excess weight loss with borderline bilirubin)	^3 (2 hyperbilirubinemia and 1 seizure from hypoparathyroidism hypocalcemia)

Main outcome and balance measures analyzed comparing each cycle versus baseline. There were no cases of symptomatic polycythemia.

*High risk infants defined as LGA, SGA, IDM, twin.

†Term is defined as 37 weeks gestational age or greater.

‡*P* < 0.05 via *t* test or Fisher exact test.

^Indicates no hematocrit screen from nursery.

Hct, hematocrit; IDM, infant of a diabetic mother; LOS, length of stay; PDSA, Plan Do Study Act through the model of improvement.

### PDSA 1 (10/1/20–10/28/20)

Of the eligible infants (N = 41), the majority were term (73%), IDM (48.8%), and SGA (26.8%). The average LOS for those remaining in the nursery to discharge was 2.1 days (SD 0.7) (t(68)=2.04, *P* = 0.04 versus baseline). Three (7.3%) infants had hematocrit lab values (*P* < 0.0001), two of which required follow-up venous hematocrits. One of the venous hematocrits was >65, but the infant remained in the nursery. In addition, there was one readmission for hyperbilirubinemia (*P* = 1.0). Unfortunately, the three hematocrits obtained had no supporting documentation.

### PDSA 2 (11/9/20–12/2/20)

Of the eligible infants (N = 30), none received a screening hematocrit (*P* < 0.0001). The majority of infants were term (76.7%), IDM (43.3%), and SGA (30%). The average LOS for those who remained in the nursery to discharge was 2.1 days (SD 0.7) (t(60)=1.7, *P* = 0.1). No infants required transfer for polycythemia, and there were two readmissions (hyperbilirubinemia and apnea/hypothermia) (*P* = 0.57). PDSA 2 was only 24 days long due to electronic medical record changes requested for PDSA3 occurring earlier than anticipated.

### PDSA 3 (12/3/20–12/30/20)

Of the eligible infants (N = 33), one received a screening hematocrit (*P* < 0.0001); the result was <65. The majority of infants were term (75.8%), IDM (48.4%), and LGA (27.2%). The average LOS for those who remained in the nursery to discharge was 2.1 days (SD 0.9) (t(58)=1.4, *P* = 0.2). No infants were transferred to the NICU for polycythemia, and there were two readmissions (hypoxia and excess weight loss) (*P* = 0.59).

### Monitoring Period (12/31/20–1/27/21)

Deimplementation of screening persisted. None of the 43 eligible infants had hematocrit laboratories. Therefore, throughout the study and monitoring period, there were no documented symptomatic polycythemia cases.

## DISCUSSION

Using improvement methodology, we successfully deimplemented polycythemia screening in our level 1 nursery, decreasing hematocrit screening in asymptomatic infants from 80% to 0% with no statistically significant increase in LOS, readmissions, or transfers. In addition, we demonstrated that simple, inexpensive measures such as education, alteration of electronic order sets, and repeated performance feedback/reminders could help eliminate low-value care.

Other unmeasured benefits of removing the screening protocol include savings in nursing time, phlebotomy time for follow-up confirmatory venous samples, cost of venous samples or any other subsequent testing, or transfer to higher-level care with additional interventions or prolonged LOS that would subsequently occur. Moreover, it may avoid the impact on breastfeeding rates that formula supplementation or transfer to the NICU would likely have.

Successful deimplementation may be challenging. The focus in medicine is often on innovation and overoptimizing existing practices.^15^ While there is often ample enthusiasm for adding something, it may be more challenging to take something away (ie, introducing glucose gel, sepsis calculator, targeted cytomegalovirus screening versus addressing the utility of polycythemia screening). According to the psychology of change, an individual perceives gains and losses differently. Deimplementation may be perceived as a loss. We subsequently assign it a greater value or worth than we may otherwise have according to the endowment effect.^4^ Moreover, confirmation bias makes us extra critical of anything that goes against our prior belief, plus we are subject to influence from prior clinical experience.^15^ It is, therefore, beneficial to frame the project in a way that highlights supplemental gains.

In our case, our success is due to (1) lack of evidence to recommend screening, (2) our evidence of the rarity of the screened-for event in our patients, as well as (3) our interdisciplinary team and the multifaceted nature of the education provided, addressing each involved professional group. Understanding the workflow within our nursery unit, our team realized it was crucial to involve the nursing staff primarily. They were usually the care members who initiated the screening process. We also made sure to frame the initiative as more than decreasing cost. We were minimizing infant needle sticks, decreasing interference with maternal-infant bonding, and saving time for staff. We invited feedback and input to ensure everyone felt safe to move forward. As many of these infants are likely to be assessed frequently for glucose checks per our hypoglycemia protocol, ample opportunity remained to note any potential symptomatic cases and intervene with a hematocrit, if indicated.

## LIMITATIONS

There are several limitations to this study. First, using PHIS data for our baseline, there was a lag between baseline and intervention; however, we could not identify any substantial changes during that time that would significantly sway practice habits away or toward screening. The lag allows us to be confident that steps during the preparation process did not affect the baseline data collected. Second, we cannot know the accuracy of the problem list for subject selection. However, we do have a clinical documentation improvement team that promotes accurate coding for optimal reimbursement. Checking the patient list twice a day should also minimize the odds of missed ICD codes or patient transfers. Third, because there was no significant break between PDSA cycles, the relative effect of each intervention alone is challenging to estimate. With each PDSA cycle, we reinforced the importance of screening any symptomatic cases and delineating desired documentation and follow-up responsibilities. This approach was for patient safety and allowed us to better differentiate symptomatic from asymptomatic infants and appropriately choose eligible charts for review. Fourth, there was a documentation deficit for obtained hematocrits, but we reviewed vital signs and all EMR notes. Finally, it is pertinent to remain aware that months of monitoring may not be sufficient to encounter a rare outcome, such as hyperviscosity from polycythemia. We only monitored for 1 month after completing the last PDSA cycle, limiting our ability to determine whether these results were sustained long-term or if unintended adverse consequences subsequently occurred. However, there is insufficient literature to determine effect size and calculate power or optimal sample size.

## CONCLUSIONS

We decreased polycythemia screening in asymptomatic infants from 80% to 0% through education, feedback, reminders, and removal of electronic health record prompts. We crafted a successful deimplementation process by identifying key drivers and a process map. Our team achieved these results without significant observed adverse effects. It is crucial that we routinely assess the utility of our practice habits or pathways/guidelines and the evidence behind them to consider management change, as residents often graduate to practice as they learn.^16,17^ As demonstrated, the use of simple low-resource interventions can enable us to make needed changes.

## ACKNOWLEDGMENTS

Dr. Priya Jain assisted Dr. Johnson as a mentor through the Future Leaders Program.

## DISCLOSURE

The authors have no financial interest to declare in relation to the content of this article.
